# ALX/FPR2 Contributes to Serum Amyloid A‐Induced Lung Neutrophil Recruitment Following Acute Ozone Exposure

**DOI:** 10.1096/fj.202402865R

**Published:** 2025-05-27

**Authors:** Michael J. Yaeger, Tyson Ngatikaura, Natali Zecchino, Katelyn Dunigan‐Russell, Hannah B. Lovins, Evangeline Schott, Grace Hutton, Brett Saunders, Yan Lin, Junfeng (Jim) Zhang, Samuel J. Cochran, Rafia Virk, R. Ian Cumming, Salik Hussain, Robert M. Tighe, Saame Raza Shaikh, Kymberly M. Gowdy

**Affiliations:** ^1^ Department of Internal Medicine Ohio State University Wexner Medical Center Columbus Ohio USA; ^2^ Nicholas School of the Environment Duke University Durham North Carolina USA; ^3^ Department of Nutrition, Gillings School of Global Public Health and School of Medicine University of North Carolina at Chapel Hill Chapel Hill North Carolina USA; ^4^ Department of Physiology, Pharmacology & Toxicology West Virginia University Morgantown West Virginia USA; ^5^ Department of Medicine Duke University Durham North Carolina USA

**Keywords:** ALX/FPR2, lung, neutrophil, ozone, serum amyloid A

## Abstract

Ozone (O_3_) is a toxic air pollutant that causes pulmonary inflammation, neutrophil recruitment, and lung injury. Part of the inflammatory response to O_3_ includes altered expression of formyl peptide receptor 2 (ALX/FPR2), a G protein‐coupled receptor expressed primarily in immune cells. ALX/FPR2 is considered either anti‐inflammatory/proresolving or proinflammatory depending on its ligands, which include lipoxin A4 or serum amyloid A (SAA). While the anti‐inflammatory/proresolving lipoxin A4 ligand has been well studied, there remains a significant knowledge gap in the interaction between proinflammatory SAA and ALX/FPR2. To date, SAA has been shown to increase neutrophil recruitment through ALX/FPR2 and is increased systemically after O_3_ exposure. However, it is unclear if pulmonary SAA signals through ALX/FPR2 during the O_3_‐induced inflammatory response. We hypothesized that ALX/FPR2‐SAA signaling is required to initiate neutrophil recruitment to the lungs following O_3_ exposure. To test this hypothesis, ALX/FPR2 wild type (FPR2^+/+^) or knockout (FPR2^−/−^) mice were exposed to filtered air (FA) or 1 ppm O_3_ for 3 h. Pulmonary inflammation was assessed 6, 24, and 48 h following O_3_ exposure. FPR2^−/−^ mice exhibited impaired neutrophil recruitment at 6 and 24 h after O_3_ exposure. In addition, FPR2^−/−^ mouse pulmonary SAA expression was significantly increased after O_3_ exposure compared to FPR2^+/+^ mice. FPR2^+/+^ mice dosed with SAA via oropharyngeal aspiration had increased pulmonary neutrophils, while neutrophils were not increased in FPR2^−/−^ mice. Taken together, these data indicate that ALX/FPR2 may contribute to SAA‐induced pulmonary neutrophilia following O_3_ exposure.

## Graphical abstract

Ozone (O_3_) is a toxic air pollutant that causes respiratory inflammation and can cause hospitalization. The goal of this study was to determine if the G protein‐coupled receptor, ALX/FPR2, is required for the initiation of inflammation in the lungs following O_3_ exposure. We determined that O_3_ exposure induces SAA_1_ and SAA_3_ production, which signal through ALX/FPR2 to recruit neutrophils to the lung.

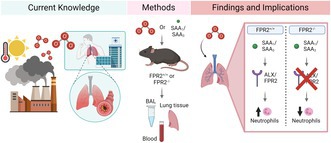

## 1 Introduction

Air pollution is the fourth highest risk factor for death and was attributed to more than 6.67 million deaths globally in 2019 [1]. Ozone (O_3_) is a major contributor to the adverse health effects from air pollution. This is in part because it induces lung inflammation/injury and increases the incidence and exacerbation of chronic lung diseases [2, 3]. Furthermore, tropospheric levels of O_3_ have increased over the last several years, which has led to increased acute O_3_ exposures [4, 5]. This is of concern because acute O_3_ exposure is correlated with increased hospitalizations and deaths, particularly in people with preexisting cardiopulmonary diseases [6, 7, 8, 9]. Therefore, it is important to define the biological mechanisms by which O_3_ contributes to lung inflammation and injury to better identify how to mitigate detrimental health effects from O_3_ exposure.

O_3_ causes lung injury partly through activating the inflammatory response [10]. When inhaled, O_3_ does not directly interact with cells, but rather interacts with the airway surface liquid where it oxidizes phospholipids, proteins, mucins, and cholesterol [2]. These O_3_‐induced modifications generate damage associated molecular patterns (DAMPs), molecular signals which are then detected by pattern recognition receptors such as toll‐like receptor 4 (TLR4). Signaling through TLR4 leads to downstream activation of transcription factors such as nuclear transcription factor‐κB (NF‐κB) which transcribe proinflammatory cytokines and chemokines [2, 11, 12, 13]. These cytokines and chemokines activate the acute phase response, including production of serum amyloid A (SAA), which then recruits innate immune cells (i.e., neutrophils) to the lungs [14, 15, 16].

We previously published that O_3_ exposure decreases pulmonary expression of the SAA receptor, ALX/FPR2 [17]. ALX/FPR2 is a 7‐pass transmembrane G protein‐coupled receptor implicated in pulmonary diseases such as asthma [18, 19], pneumosepsis [20], and fibrosis [21]. ALX/FPR2 is expressed primarily on leukocytes and airway epithelial cells and has many ligands that impart differing biological responses [22]. For example, in addition to binding with the proinflammatory SAA, anti‐inflammatory/proresolving lipid metabolites termed specialized proresolving mediators (SPMs)—such as lipoxin A4 (LXA_4_) and resolvin D1 (RvD_1_)—signal through ALX/FPR2 [23, 24, 25]. The biased agonism of ALX/FPR2 is still unclear; however, it has been suggested these ligand‐dependent effects are influenced by ALX/FPR2 heterodimerizing with proinflammatory FPR1 or FPR3, or homodimerizing to detect anti‐inflammatory ligands [26]. Because of ALX/FPR2's wide range of bioactivity, it is important to consider both its anti‐inflammatory and proinflammatory aspects. Presently, it is unclear if SAA signals through ALX/FPR2 during the pulmonary inflammatory response to O_3_. Therefore, we hypothesized that ALX/FPR2‐SAA signaling is required to initiate neutrophil recruitment in the lungs following O_3_ exposure by detecting SAA. In this study, loss of ALX/FPR2 reduced neutrophil recruitment to the lung following O_3_ exposure. Neutrophil recruiting chemokines were not disrupted in FPR2^−/−^ mice and the SPM response was unaltered. However, FPR2^−/−^ mice had significantly increased SAA expression in the lung after O_3_ exposure. Exogenous SAA administration caused significant neutrophil recruitment to the lungs only in FPR2^+/+^ mice, but not FPR2^−/−^ mice. Overall, these data indicate that ALX/FPR2 is required for SAA to recruit neutrophils to the lung following O_3_ exposure.

## 2 Materials and Methods

### 2.1 Animals

Male ALX/FPR2 wild type (FPR2^+/+^) and ALX/FPR2 knockout (FPR2^−/−^) mice, 8–12 weeks old, were used for this study. Given the known role of sex as well as the estrous cycle on the O_3_‐induced inflammatory responses, only males were used [27]. These mice were generated by the UNC Chapel Hill Animal Models Core using CRISPR‐Cas9 targeting the 5′ and 3′ flanking regions of ALX/FPR2 exon 2 as previously described [28]. Mice were bred in‐house for experiments, and genotypes were confirmed via PCR and gel electrophoresis. All experiments were performed in accordance with the Animal Welfare Act and the U.S. Public Health Service Policy on Humane Care and Use of Laboratory Animals after review by the Animal Care and Use Committees of The Ohio State University.

### 2.2 Murine In Vivo Exposure

FPR2^+/+^ and FPR2^−/−^ mice were exposed inside a modified metal Hinner's chamber to filtered air (FA) or 1 ppm of O_3_ for 3 h. This exposure mimics what a human would experience on an ‘O_3_ action day’ [29, 30]. O_3_ was generated and measured as previously described and temperature and humidity were monitored continuously [17]. Mice were euthanized 6, 24, or 48 h after exposure with an intraperitoneal injection of a ketamine (100 mg/kg) and xylazine (10 mg/kg) mixture as previously described [31]. For CXCL1 experiments, FPR2^+/+^ and FPR2^−/−^ mice were anesthetized with isoflurane and dosed with 0.5 μg/mouse via oropharyngeal aspiration (OA) in 50 μL of CXCL1 (R&D Systems, MN, USA) or vehicle control (PBS). In additional experiments, FPR2^+/+^ and FPR2^−/−^ mice were anesthetized with isoflurane and dosed with 10 μg/mouse via OA in 50 μL of recombinant mouse SAA_1_ (< 0.1 EU/μg; R&D Systems, MN, USA), recombinant mouse SAA_3_ (< 1 EU/μg; Cusabio, TX, USA), or vehicle control (PBS). Mice were then euthanized 4 h after dosing with CXCL1 as previously described [32], and 24 h after dosing with SAA.

### 2.3 Bronchoalveolar Lavage Fluid (BALF) Collection and Analysis

The right lung was lavaged with three separate volumes of 26.25 mL/kg of PBS as previously described [27]. BALF was then centrifuged at 460× *g* for 6 min at 4°C. Total protein was measured in BALF supernatant using the bicinchoninic acid (BCA) Protein Assay Kit (Thermo Fisher Scientific, MA, USA). Cellular differentials of cells collected in BALF were performed as previously described [27]. Additional BALF supernatant was concentrated with Amicon Ultra Centrifugal Filters (MilliporeSigma, MA, USA) before measuring cytokines and chemokines by MESO QuickPlex analysis (Mesoscale Discovery, MD, USA) per the manufacturer's instructions. Cytokines and chemokines measured in BALF supernatant included: C–C motif ligand 2 (CCL2), C–X–C motif ligand 2 (CXCL2), C–X–C motif ligand 1 (CXCL1), interleukin‐6 (IL‐6), interleukin‐1β (IL‐1β), and tumor necrosis factor‐α (TNF‐α).

### 2.4 Plasma SAA ELISA

Blood was collected by cardiac puncture with a 25 G needle and transferred to a dipotassium ethylenediaminetetraacetic acid (K_2_EDTA) plasma tube (BD Biosciences, NJ, USA). The collected blood was centrifuged at 200× *g* for 10 min at 4°C. Plasma supernatant was then collected and stored at −80°C until analysis. SAA concentrations in plasma were analyzed by ELISA using a mouse SAA quantikine ELISA kit (R&D Systems, MN, USA) per the manufacturer's instructions.

### 2.5 MPO Assay

Unperfused left lung lobes were collected immediately following euthanasia and snap frozen in liquid nitrogen. Frozen lung tissue was then homogenized via bead homogenization in 200 μL of assay buffer for MPO activity (Abcam, Cambridge, United Kingdom). Lung tissue homogenate was then centrifuged at 13 000× *g* for 10 min, and the supernatant was collected. BCA protein assay (Thermo Fisher Scientific, MA, USA) was performed on the homogenate supernatant, and then the MPO assay was performed on the supernatant per the manufacturer's instructions.

### 2.6 RNA Isolation and Quantitative Polymerase Chain Reaction (qPCR)

Left lung lobes were collected immediately after euthanasia, sealed in a cryotube, and snap frozen in liquid nitrogen until RNA isolation. Lung tissue was homogenized, and RNA was isolated using a Qiagen RNeasy Mini Kit (Fisher Scientific, NH, USA) according to the manufacturer's instruction. RNA was quantified using a NanoDrop 1000 Spectrophotometer (Thermo Fisher Scientific, MA, USA) or SpectraMax iD3 Multi‐Mode Microplate Reader (VWR, PA, USA). cDNA was synthesized from RNA using the RevertAid First Strand cDNA Synthesis Kit per the manufacturer's instructions (Thermo Fisher, MA, USA). Real‐time qPCR was performed with Taqman PCR Mix (Applied Biosystems, MA, USA) in the HT7900 ABI sequence Detection System (Applied Biosystems, MA, USA) using predesigned primers (Applied Biosystems, MA, USA). Fold changes in expression of mRNA were calculated using Ct values and the 2^−ΔΔCt^ method. Samples were normalized to 18S as previously described [33]. Taqman primers used for this research were 18S (Mm03928990_g1), SAA_1_ (Mm00656927_g1), SAA_3_ (Mm00441203_m1), and ALX/FPR2 (Mm00484464_s1) (Thermo Fisher Scientific, MA, USA).

### 2.7 Flow Cytometry

Blood, BALF, and lung tissue were collected for extracellular staining and flow cytometry analysis. Blood was collected by cardiac puncture with a 25 G needle into a K_2_EDTA tube. Then, 50 μL of blood was mixed with Fc blocking solution (antimouse CD16/32; clone 93; BioLegend, CA, USA) for 15 min, followed by incubation with an antibody mix solution for 20–30 min (antibodies listed in Table 1). Then, red blood cells (RBCs) were lysed with 1X BD's RBC lysing solution (BD Biosciences, NJ, USA), and the remaining cells were suspended in FACS buffer (PBS, 3% FBS, 10 mM EDTA, 10 mM HEPES). For BALF flow cytometry, the whole lung was lavaged with three separate volumes of 35 mL/kg of HBSS. BALF was then centrifuged at 460× *g* for 6 min at 4°C, the supernatant was aspirated, and RBCs were lysed with ACK RBC lysis buffer (0.17 M NH_4_Cl, 10 mM KHCO_3_, 250 μM EDTA in diH_2_O, pH 7.5) for 1 min before ending the lysis reaction with 4 mL of HBSS. The cells were then centrifuged at 460× *g* for 6 min at 4°C, the supernatant was aspirated, the cells were resuspended in 1 mL HBSS +1% FBS, and cellular counts were performed on a hemacytometer. The cells were then stained with 10 mM 2′,7′‐dichlorodihydrofluorescein diacetate (H2DCFDA; Thermo Fisher Scientific, MA, USA)—which converts to 2′,7′‐dichlorofluorescein (DCF) upon oxidation—for 20 min, followed by blocking with Fc blocking solution (antimouse CD16/32; clone 93; BioLegend, CA, USA), followed by staining with antibody mix solution for 30 min. Flow cytometry on blood and BALF samples was performed on the Cytek Northern Lights (Cytek Biosciences, CA, USA) spectral flow cytometer, and analysis was performed in FlowJo software (BD Life Sciences, NJ, USA). For lung tissue flow cytometry, lungs were perfused by injecting up to 10 mL of PBS into the right atrium/ventricle using a 25 G needle. The lungs were then instilled through the trachea with 2–3 mL digest buffer (5 mg/mL collagenase +0.4 mg/mL DNase +5% FBS). Filled lungs were excised and placed in a 50 mL tube with 5–10 mL additional digest buffer and incubated in a hot water bath at 37°C for 30 min, with vortexing every 8–10 min. The enzymatic reaction was stopped by filling the rest of the tube with PBS, and digested tissue was filtered through a 70 μm cell strainer. The single cell suspension was then centrifuged at 250× *g* for 6–8 min at 4°C, and RBCs were lysed with 3 mL of ACK RBC lysis buffer for 3–5 min. The single cell suspension was then pelleted and resuspended for cell counts. Approximately 10^7^ cells were stained with zombie UV (1:500; BioLegend) for 15–20 min, washed, and then fixed with 1.5% paraformaldehyde and stored at 4°C. For antibody staining, cells were incubated with Fc blocking solution (BioLegend, CA, USA) for 7–10 min and then stained with the antibody mix solution for 30 min (antibodies listed in Table 1) as previously described by Yu et al. [34]. Flow cytometry was performed on an LSR Fortessa X‐20, and data analysis was performed in FlowJo.

**TABLE 1:** Flow cytometry antibodies.

Antibody	Fluorophore	Dilution	Clone	Vendor
Blood				
CD45	APC/Cy7	1:100	30‐F11	BioLegend
CD11b	BV711	1:100	M1/70	BioLegend
Ly6g	BV421	1:100	1A8	BioLegend
BALF				
CD45	APC/Cy7	1:100	30‐F11	BioLegend
Ly6g	APC	1:100	S19019G	BioLegend
Lung tissue				
CD206	FITC	1:400	C068C2	BioLegend
SiglecF	PE/CF594	1:1500	E50‐2440	BD Biosciences
Ly6G	AF700	1:200	1A8	BioLegend
CD11b	APC/Cy7	1:150	M1/70	BioLegend
CD64	BV421	1:200	X54‐5/7.1	BioLegend
CD45	BV605	1:500	30‐F11	BioLegend
IA/IE	BV650	1:1500	M5/114.15.2	BioLegend
CD24	BV711	1:800	M1/69	BioLegend
CD11c	BV785	1:150	N418	BioLegend
Zombie UV		1:1000		BioLegend

### 2.8 Liquid Chromatography‐Mass Spectrometry

All standards and internal standards used for reverse phase high performance liquid chromatography (HPLC) tandem mass spectrometry (LC–MS/MS) analysis of lipid mediators were purchased from Cayman Chemical (Ann Arbor, Michigan). All HPLC solvents and extraction solvents were HPLC grade. Left lung tissue was homogenized, and lipid mediators were isolated as previously described [27]. The samples were analyzed immediately or frozen at −70°C until analysis. Isolated lipid metabolites were analyzed by LC–MS/MS as previously described [17]. Briefly, the Agilent 6490 triple quadrupole mass spectrometer in negative ionization mode was used to detect lipid mediators. The Agilent Masshunter Quantitative Analysis software was used to calculate calibration curves, which were then used to calculate pg/mg of wet tissue. All standards were purchased from Cayman Chemical. All nondetectable samples were assigned a value of zero for statistical analysis.

### 2.9 Statistical Analysis

Data points were pooled from two separate experiments and are expressed as mean ± standard error of the mean (SEM). Data were analyzed using two‐way ANOVA followed by Šídák's multiple comparisons test post hoc to correct for multiple comparisons. A value of *p* < 0.05 was considered significant. For Figure 5A–F, lipidomic data were normalized by min‐max scaling following the equation: $x_{\text{scaled}}=\frac{x-x_{\min}}{x_{\max}-x_{\min}}$ [35]. These data were normalized to better assess changes in the overall lipidome without individual high‐concentration lipids (e.g., 12‐HETE, 14‐HDoHE, 12‐HEPE) disproportionately influencing the results.

## 3 Results

### 3.1 ALX/FPR2 Expression in the Lung is Altered by O_3_ Exposure

**FIGURE 1:**
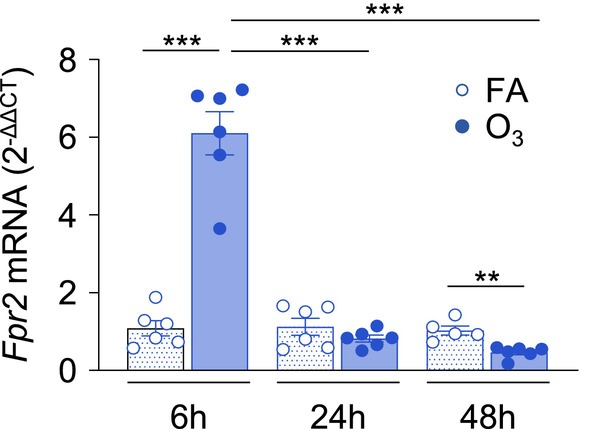
Lung ALX/FPR2 expression in the lung is altered by ozone exposure. ALX/FPR2 wild type (FPR2^+/+^) male mice 8–12 weeks old were exposed to filtered air (FA) or 1 ppm ozone (O_3_) for 3 h, and necropsied 6, 24, or 48 h after the start of the exposure. Lung tissue was collected for real‐time PCR analysis. **p < 0.01, ***p < 0.001, *n* = 5–6/group.

To first assess how ALX/FPR2 changes over the course of the inflammatory response to O_3_ exposure, *Fpr2* gene expression in lung tissue was measured at 6, 24, and 48 h after exposure (Figure 1). At 6 h, *Fpr2* expression was increased in O_3_‐exposed mice compared to FA. Then, at 24 h, *Fpr2* was not statistically altered in O_3_‐exposed mice compared to FA. Finally, at 48 h, *Fpr2* was significantly decreased in O_3_‐exposed mice compared to FA. Overall, these findings show that *Fpr2* gene expression in lung tissue is altered by O_3_ exposure in a time‐dependent manner.

### 3.2 ALX/FPR2 Contributes to the Initiation of O_3_ ‐Induced Pulmonary Inflammation

**FIGURE 2:**
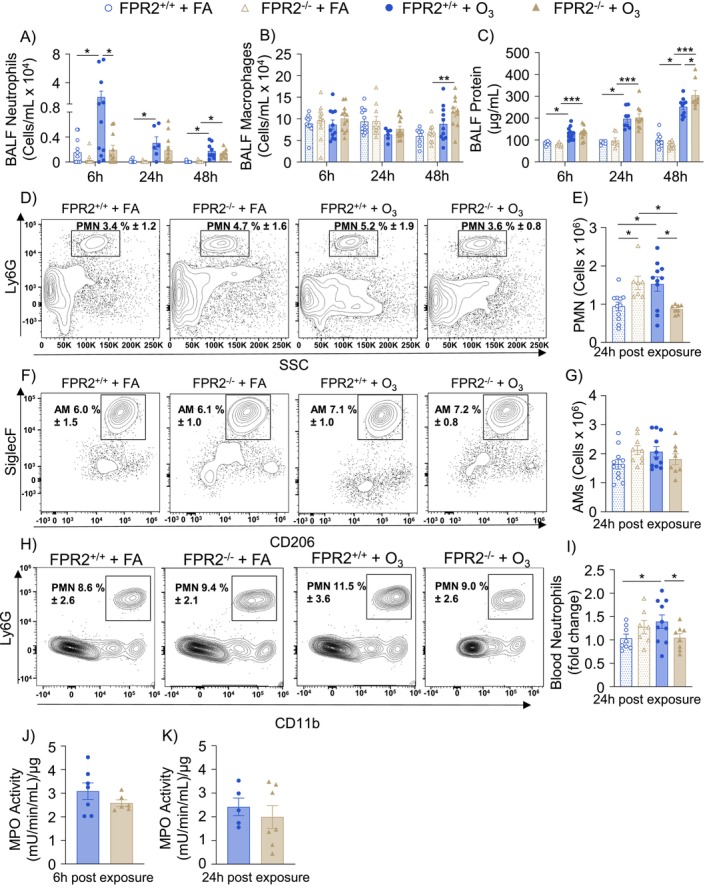
ALX/FPR2 contributes to the initiation of ozone‐induced pulmonary inflammation. ALX/FPR2 wild type (FPR2^+/+^) or ALX/FPR2 knockout (FPR2^−/−^) male mice 8–12 weeks old were exposed to filtered air (FA) or 1 ppm ozone (O_3_) for 3 h and necropsied 6, 24, or 48 h after the start of the exposure. (A) Bronchoalveolar lavage fluid (BALF) was collected for cellular differentials of neutrophils, and (B) macrophages, and for (C) protein analysis. Lung tissue was collected for flow cytometry analysis of (D, E) neutrophils, and (F, G) alveolar macrophages (AMs). (H, I) Blood was collected for flow cytometry analysis of neutrophils. Lung tissue was collected for MPO activity analysis at (J) 6 h and (K) 24 h following O_3_ exposure. MPO activity is reported in milliunits (mU) per minute of enzymatic reaction per milliliter (mL) of reaction volume per microgram (μg) of total protein in the reaction well. *p < 0.05, ***p < 0.001, *n* = 6–13/group.

Given that *Fpr2* expression in the lung was changed following O_3_ exposure, we sought to evaluate the impact of ALX/FPR2 on immune cell recruitment and lung injury after O_3_ exposure. FPR2^−/−^ and FPR2^+/+^ mice were exposed to whole‐body FA or 1 ppm O_3_ for 3 h and necropsied 6, 24, or 48 h following exposure. At 6 h following O_3_ exposure, airspace neutrophils were increased in FPR2^+/+^ but not in FPR2^−/−^ mice and were significantly decreased in FPR2^−/−^ compared to FPR2^+/+^ mice (Figure 2A). Then 24 h following O_3_ exposure, airspace neutrophilia continued to be increased compared to FA groups in FPR2^+/+^ mice, but not in FPR2^−/−^ mice (Figure 2A). At 48 h following O_3_ exposure, BALF neutrophils were increased in both FPR2^+/+^ and FPR2^−/−^ mice with no differences between genotypes (Figure 2A). O_3_ did not alter numbers of airspace macrophages at 6 or 24 h; however, O_3_ significantly increased BALF macrophages in FPR2^−/−^ mice at 48 h after exposure (Figure 2B). O_3_ exposure increased microvascular and alveolar epithelial permeability, as measured via BALF protein, at all timepoints post exposure. At 6 and 24 h following O_3_, BALF protein was not different between FPR2^+/+^ and FPR2^−/−^ mice (Figure 2C). At 48 h post exposure, O_3_‐exposed FPR2^−/−^ mice had statistically increased BALF protein compared to FPR2^+/+^ mice (Figure 2C). These findings suggest that ALX/FPR2 contributes to increased airspace neutrophils during the initiation of inflammation as well as protects against lung injury following O_3_ exposure at later timepoints.

### 3.3 ALX/FPR2 Facilitates O_3_ ‐Induced Pulmonary Neutrophil Recruitment

Reduced airspace neutrophilia in FPR2^−/−^ mice could result from impaired neutrophil trafficking from the interstitium to the airspace. To investigate this, flow cytometry was performed on perfused and digested lung tissue to evaluate whole lung neutrophilia. Flow cytometry indicated that neutrophils were increased in the lung tissue of FPR2^−/−^ mice 24 h following FA exposure when compared to FPR2^+/+^ mice (Figure 2D,E). 24 h following O_3_ exposure, neutrophils were increased in FPR2^+/+^ mice and were significantly decreased in FPR2^−/−^ mice compared to FPR2^+/+^ mice (Figure 2D,E). Alveolar macrophages were also evaluated by flow cytometry, which were not altered by exposure and/or genotype (Figure 2F,G). Since neutrophils were decreased in both the airspace and the whole lung, these data suggest that ALX/FPR2 influences neutrophil recruitment to the lung. To understand whether altered pulmonary neutrophil migration results from reduced systemic levels, blood neutrophils were measured by flow cytometry. O_3_ significantly increased neutrophilia in the blood of FPR2^+/+^ mice but did not alter neutrophils in the blood of FPR2^−/−^ mice (Figure 2H,I). Moreover, among O_3_‐exposed animals, blood neutrophils were significantly decreased in FPR2^−/−^ mice compared to FPR2^+/+^ mice (Figure 2E,F). This suggests reduced circulating neutrophil levels, perhaps due to reduced chemotaxis from the bone marrow. Lung tissue myeloperoxidase (MPO) was also measured to determine if loss of ALX/FPR2 disrupts neutrophil function in addition to chemotaxis following O_3_ exposure. At both 6 and 24 h following O_3_ exposure, MPO was not significantly altered between FPR2^+/+^ and FPR2^−/−^ mice (Figure 2J,K). Therefore, the effect of ALX/FPR2 on neutrophils appears to be limited to their recruitment following O_3_ exposure.

### 3.4 FPR2 ^−/−^ Neutrophils Retain Chemotactic and Reactive Oxygen Species Producing Functions

**FIGURE 3:**
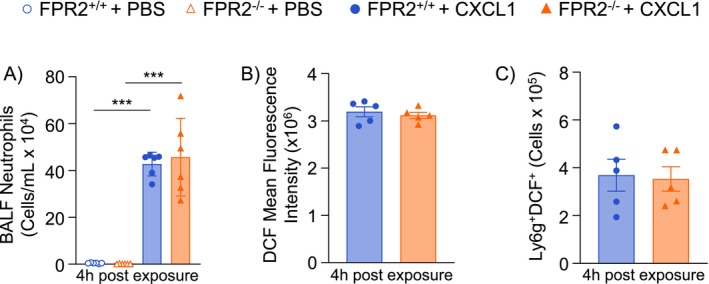
FPR2^−/−^ neutrophils retain chemotactic and reactive oxygen species producing function. ALX/FPR2 wild type (FPR2^+/+^) or ALX/FPR2 knockout (FPR2^−/−^) male mice 8–12 weeks old were dosed by oropharyngeal aspiration (OA) with PBS or 0.5 μg/mouse CXCL1 and then necropsied 4 h after exposure. Bronchoalveolar lavage fluid (BALF) was collected for (A) cellular differentials of neutrophils, and (B/C) flow cytometry analysis of reactive oxygen species using a 2′,7′‐dichlorofluorescein (DCF) dye. ***p < 0.001, *n* = 5–6/group.

To then identify if neutrophils lacking ALX/FPR2 had reduced chemotactic responsiveness, mice were dosed by OA with CXCL1, a neutrophil‐recruiting chemokine. Following CXCL1 dosing, both FPR2^+/+^ and FPR2^−/−^ mice had increased airspace neutrophilia with no differences between genotypes (Figure 3A). This suggests that FPR2^−/−^ neutrophils can still be chemotactically recruited to the lungs and therefore the effect of ALX/FPR2 on pulmonary neutrophil chemotactic signaling is indirect. To identify if the loss of ALX/FPR2 disrupts neutrophil function, neutrophils from the BALF were also evaluated for reactive oxygen species (ROS), using the 2′,7′‐dichlorodihydrofluorescein diacetate (H_2_DCFDA) dye that converts to the highly fluorescent 2′,7′‐dichlorofluorescein (DCF) upon oxidation. Neutrophils from FPR2^−/−^ mice had the same mean fluorescence intensity for DCF, as well as the same number of Ly6G^+^DCF^+^ cells as FPR2^+/+^ mice when stimulated with CXCL1 by OA (Figure 3B,C). This indicates FPR2^−/−^ neutrophils retain their ROS production function.

### 3.5 Inflammatory Signaling Is Not Disrupted With Loss of ALX/FPR2 Following O_3_ Exposure

**FIGURE 4:**
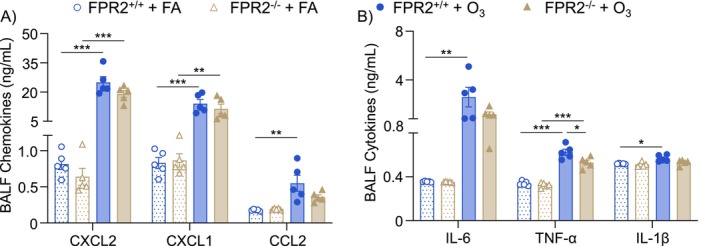
Initiation of inflammatory signaling is not disrupted with loss of ALX/FPR2 following ozone exposure. ALX/FPR2 wild type (FPR2^+/+^) or ALX/FPR2 knockout (FPR2^−/−^) male mice 8–12 weeks old were exposed to filtered air (FA) or 1 ppm ozone (O_3_) for 3 h and necropsied 6 h after the start of the exposure. Bronchoalveolar lavage fluid (BALF) was collected for mesoscale analysis of (A) chemokines and (B) cytokines. *p < 0.05, **p < 0.01, ***p < 0.001, *n* = 5/group.

To define potential reasons why neutrophils are not effectively recruited to the lungs in FPR2^−/−^ mice after O_3_ exposure, airspace cytokines and chemokines were measured in the BALF. CXCL1 and CXCL2 (neutrophil recruiting chemokines) were increased in both FPR2^+/+^ and FPR2^−/−^ mice with no differences between genotypes 6 h after O_3_ exposure (Figure 4A). CCL2 (monocyte recruiting chemokine) was statistically increased in FPR2^+/+^ mice but not in FPR2^−/−^ mice after O_3_ exposure (Figure 4A). IL‐6 and IL‐1β were increased only in FPR2^+/+^ mice after O_3_ exposure (Figure 4B). TNF‐α was also increased in both FPR2^+/+^ and FPR2^−/−^ mice but decreased in FPR2^−/−^ mice compared to FPR2^+/+^ mice after O_3_ exposure (Figure 4B). Thus, loss of ALX/FPR2 influences the production of select proinflammatory cytokines and chemokines but not neutrophil‐recruiting chemokines. Given that neutrophil‐recruiting chemokines were unaffected, this suggests a role for other O_3_‐induced soluble mediators.

### 3.6 Loss of ALX/FPR2 Does Not Influence Pulmonary Oxylipins

**FIGURE 5:**
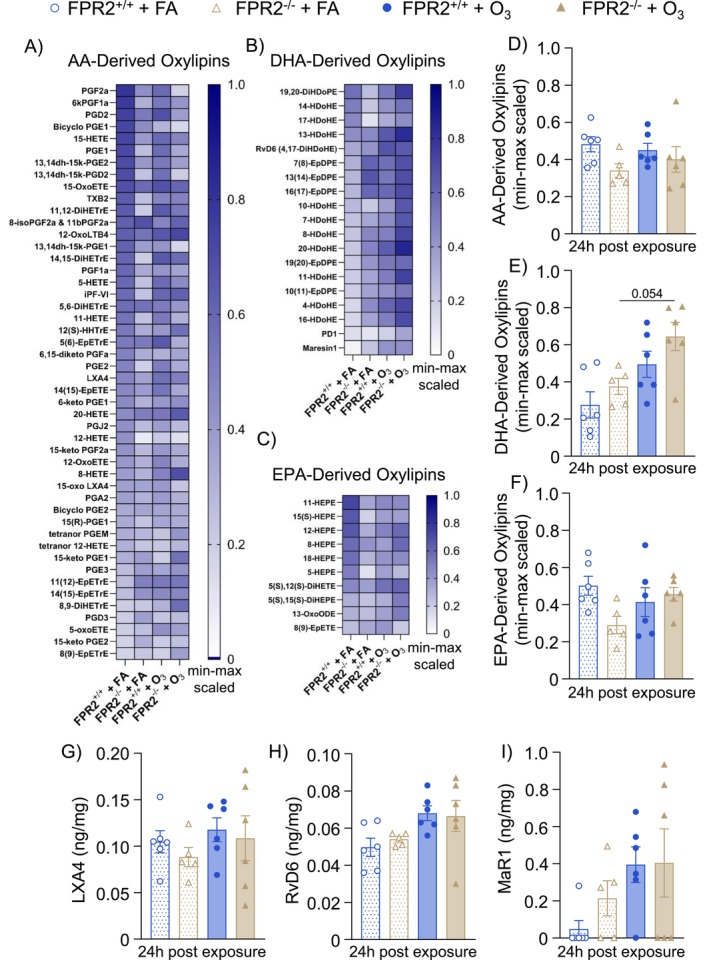
Loss of ALX/FPR2 does not influence pulmonary specialized pro‐resolving mediators. ALX/FPR2 wild type (FPR2^+/+^) or ALX/FPR2 knockout (FPR2^−/−^) male mice 8–12 weeks old were exposed to filtered air (FA) or 1 ppm ozone (O_3_) for 3 h and necropsied 24 h after the start of the exposure. Lung tissue was collected for LC–MS/MS analysis of oxylipins. Data were normalized by min‐max scaling. Heatmap of normalized values for (A) arachidonic acid (AA)‐derived oxylipins, (B) docosahexaenoic acid (DHA)‐derived oxylipins, (C) eicosapentaenoic acid (EPA)‐derived oxylipins. Average of normalized values for (D) AA‐derived oxylipins, (E) DHA‐derived oxylipins, (F) EPA‐derived oxylipins. Tissue concentrations of selected specialized proresolving mediators (G) lipoxin A4 (LXA4), (H) resolvin D6 (RvD6), and (I) maresin 1 (MaR1). *n* = 5–6/group.

Neutrophil recruitment can also be influenced by oxylipins derived from n‐6 (typically proinflammatory eicosanoids) and n‐3 (typically anti‐inflammatory/proresolving SPMs) polyunsaturated fatty acids [36, 37, 38]. Furthermore, ALX/FPR2 is a known receptor for SPMs LXA4 and RvD1. Therefore, oxylipins were measured in lung tissue by targeted LC–MS/MS to determine whether they were altered by the loss of ALX/FPR2, which may have led to changes in neutrophil recruitment following O_3_ exposure. Separating oxylipins by parent fatty acids, arachidonic acid (AA; 20:4 n‐6), docosahexaenoic acid (DHA; 22:6 n‐3), and eicosapentaenoic acid (EPA; 20:5 n‐3) derived oxylipins were not statistically altered by exposure or genotype. However, DHA‐derived oxylipins appeared to nonstatistically increase after O_3_ exposure, particularly in FPR2^−/−^ mice (Figure 5A–F). Furthermore, there were no changes in the ALX/FPR2 ligand, LXA4 (Figure 5G), or other SPMs such as RvD6 (Figure 5H) or MaR1 (Figure 5I) 24 h after FA or O_3_ exposure. The other SPM ligand for ALX/FPR2, RvD1, was below the limit of detection and unable to be quantified. All detected oxylipins are presented in Table S1. Overall, the loss of ALX/FPR2 signaling does not likely exert an effect on neutrophilia by disrupting oxylipin concentrations.

### 3.7 SAA Expression and Production Are Disrupted With Loss of ALX/FPR2

**FIGURE 6:**
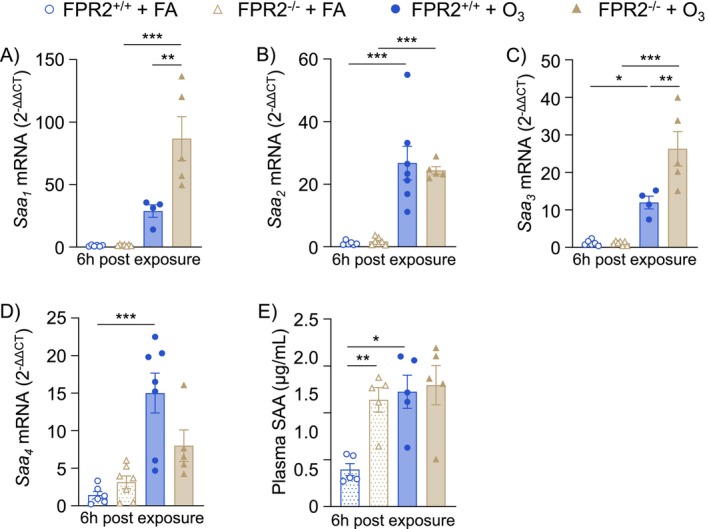
Serum amyloid A expression and production is disrupted with loss of ALX/FPR2. ALX/FPR2 wild type (FPR2^+/+^) or ALX/FPR2 knockout (FPR2^−/−^) male mice 8–12 weeks old were exposed to filtered air (FA) or 1 ppm ozone (O_3_) for 3 h and necropsied 6 h after the start of the exposure. Lung tissue was collected for real‐time PCR of (A) *Saa*_*1*_, (B) *Saa*_*2*_, (C) *Saa*_*3*_, (D) *Saa*_*4*_. (E) Plasma was collected for ELISA analysis of total SAA. *p < 0.05, **p < 0.01, ***p < 0.001, *n* = 4–7/group.

With no changes in neutrophil recruiting chemokines or proresolving SPMs, SAA—a pro‐inflammatory ligand for ALX/FPR2—was investigated. There are three members of the SAA family expressed in mice that function in acute phase responses (SAA_1–3_), and a fourth SAA, SAA_4_, is constitutively expressed. SAA_1_ and SAA_2_ are homologous and mostly produced in the liver but can also be produced extrahepatically, while SAA_3_ is mostly expressed extrahepatically [39, 40]. O_3_ exposure induced pulmonary *Saa*_*1*_, *Saa*_*2*_, *Saa*_*3*_, and *Saa*_*4*_ expression in FPR2^+/+^ mice (Figure 6A–D). In FPR2^−/−^ mice, *Saa*_*1*_ and *Saa*_*3*_ were significantly increased, and *Saa*_*2*_ was not different compared to FPR2^+/+^ mice after O_3_ exposure (Figure 6A–C). *Saa*_*4*_ was not increased in FPR2^−/−^ mice after O_3_ exposure (Figure 6D). Plasma concentrations of total SAA were also measured by ELISA. Plasma SAA was significantly increased in FA exposed FPR2^−/−^ mice compared to FA exposed FPR2^+/+^ mice (Figure 6E). FPR2^+/+^ mice had increased plasma SAA after O_3_ exposure compared to FA, while SAA concentrations remained elevated in FPR2^−/−^ mice, near equivalent to concentrations in FPR2^+/+^ mice, after O_3_ exposure (Figure 6E). Thus, pulmonary and systemic SAA regulation is disrupted by loss of ALX/FPR2 before and after O_3_ exposure.

### 3.8 SAA_1_ Contributes to Neutrophil Recruitment Through ALX/FPR2

**FIGURE 7:**
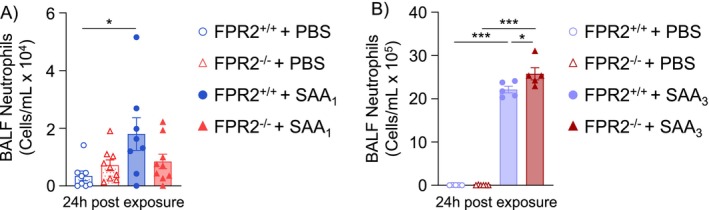
Serum amyloid A contributes to neutrophil recruitment through ALX/FPR2. ALX/FPR2 wild type (FPR2^+/+^) or ALX/FPR2 knockout (FPR2^−/−^) male mice 8–12 weeks old were dosed with PBS or 10 μg/mouse SAA_1_ by oropharyngeal aspiration (OA) and necropsied 24 h after dosing. (A) Bronchoalveolar lavage fluid (BALF) was collected for cellular differentials of neutrophils. (B) FPR2^+/+^ or FPR2^−/−^ male mice 8–12 weeks old were dosed with PBS or 10 μg/mouse SAA_3_ by OA and necropsied 24 h after dosing. Bronchoalveolar lavage fluid (BALF) was collected for cellular differentials of neutrophils. *p < 0.05, **p < 0.01, ***p < 0.001, *n* = 5–6/group.

In this study, changes in pulmonary and systemic SAA correlated with inhibited pulmonary neutrophil recruitment following O_3_ exposure. Because SAA‐ALX/FPR2 signaling leads to increased neutrophil recruitment, the loss of ALX/FPR2 may result in reduced signaling from SAA and subsequently reduced airspace neutrophil recruitment following O_3_ exposure. To test this, mice were dosed via OA with recombinant mouse SAA_1_ or SAA_3_. FPR2^+/+^ mice dosed with SAA_1_ had increased BALF neutrophils whereas FPR2^−/−^ mice dosed with SAA_1_ did not have increased BALF neutrophils (Figure 7A). Both FPR2^+/+^ and FPR2^−/−^ mice dosed with SAA_3_ had increased BALF neutrophils, and neutrophils were significantly increased in FPR2^−/−^ mice compared to FPR2^+/+^ mice (Figure 7B). These data imply that SAA_1_, but not SAA_3_, signals through ALX/FPR2 to induce neutrophil recruitment to the lung.

## 4 Discussion

The data presented here indicate that ALX/FPR2 is required to initiate neutrophil recruitment in the lungs following O_3_ exposure. We identified that ALX/FPR2 is not required for the pulmonary production of neutrophil‐recruiting chemokines or SPMs. However, pulmonary and systemic SAA were significantly altered in mice lacking ALX/FPR2, suggesting a disruption in the ALX/FPR2–SAA signaling axis. Additionally, ALX/FPR2‐deficient mice were insensitive to SAA_1_‐mediated lung neutrophil recruitment, indicating that SAA_1_ requires ALX/FPR2 to recruit lung neutrophils. These findings reveal a novel role for SAA–ALX/FPR2 signaling during O_3_‐induced lung inflammation.

As a receptor for two SPM species, ALX/FPR2 has primarily been studied for its anti‐inflammatory and/or proresolving roles [27]. However, there is also evidence for ALX/FPR2 signaling in proinflammatory pathways [28]. The present study focuses on the proinflammatory role of ALX/FPR2 as highlighted by disrupted neutrophil recruitment (Figure 2) and altered SAA production (Figure 6) in FPR2^−/−^ mice. SAA is a family of four proteins with similar functions (SAA_1‐4_) and is classically a biomarker of inflammation [25]. SAA_1_ and SAA_2_ have similar amino acid sequences in both mice and humans and are mostly produced in the liver during acute phase responses [41]. SAA_3_ is also expressed in both mice and humans (although possibly as a pseudogene in humans as discussed later), mostly in nonhepatic cells such as epithelial and hematopoietic cells during the acute phase response [42]. SAA_4_ is constitutively expressed and is generally considered not to be involved in the acute phase response [40]. The SAA proteins are generally grouped together when discussing the acute phase response, but our work and others' have indicated there are significant differences between these family members [43, 44]. For example, SAA_1_/SAA_2_ are downregulated while SAA_3_ is upregulated during amyloidosis [43]. Furthermore, SAA_3_ was reported to signal specifically through the TLR4/MD‐2 pathway while SAA_1_ favors signaling through FPR1 or ALX/FPR2 [44]. SAA_1_ has been reported to induce neutrophil chemotaxis although this mechanism has not previously been defined during O_3_ responses [45, 46]. Furthermore, there are several studies indicating “SAA” (subtype not specified) induces neutrophil recruitment [47, 48, 49] while, to the best of our knowledge, the neutrophil chemotactic ability of SAA_3_ has only been observed by association [50]. In the context of O_3_, previous studies have reported that *Saa*_*1*_ and *Saa*_*2*_ expression in liver tissue and total SAA protein in the blood are increased following rodent exposure [51]. In addition, similar to our results, *Saa*_*3*_ was significantly increased in lung tissue of O_3_ exposed mice [52]. Therefore, SAAs are present following O_3_ exposure; however, it is unclear if a particular SAA family member is dominant in this response or if this is dependent on ALX/FPR2.

In this study, pulmonary expression of all *Saa* genes was increased after O_3_ exposure. Interestingly, O_3_‐induced lung *Saa*_*1*_ and *Saa*_*3*_ expressions were higher in the FPR2^−/−^ mice compared to FPR2^+/+^ mice, while *Saa*_*2*_ did not differ in FPR2^−/−^ mice compared to FPR2,^+/+^ and *Saa*_*4*_ did not increase after O_3_ exposure in FPR2^−/−^ mice (Figure 6). This suggests ALX/FPR2 differentially influences SAA family members' transcription. SAA transcription is induced by pro‐inflammatory cytokines such as IL‐6, which binds the receptor gp130 to induce STAT3 transcription of SAA_1_ [53]. SAA_1_ transcription is also regulated by AP‐2 and NF‐κB [54]. In addition, IL‐6 or IL‐1β can synergistically enhance NF‐κB interaction with the SAA_3_ promoter [55, 56]. In this study, IL‐6 and IL‐1β were only statistically increased after O_3_ exposure in FPR2^+/+^ mice (Figure 4B). This suggests that the loss of ALX/FPR2 may have reduced cytokine signaling associated with *Saa*_*1*_ and *Saa*_*3*_ transcription. In the context of O_3_, it is unclear how IL‐6/IL‐1β concentrations relate to SAA transcripts; however, the loss of ALX/FPR2 could disrupt temporal regulation of cytokine and/or SAA signaling. There are also potential feedback mechanisms that regulate SAA transcription [39]. It has been proposed that SAA leads to recruitment and enhanced survival of myeloid‐derived suppressor cells (MDSCs) which downregulate IL‐1β/IL‐6 production and therefore inhibit further SAA transcription [39]. If the loss of ALX/FPR2 disrupts MDSC recruitment and prevents the downregulation of SAA, then this may account for the overexpression of *Saa*_*1*_ and *Saa*_*3*_ observed in FPR2^−/−^ mice (Figure 6A,C). Further research is needed to better understand how ALX/FPR2 regulates pulmonary SAA transcription and translation.

We also investigated how ALX/FPR2 influences how the lungs respond to increased SAA. Our findings show that OA administration of SAA_1_ and SAA_3_ resulted in differential neutrophil recruitment responses (Figure 7). SAA_3_ induced a much more robust neutrophil response when compared to SAA_1_, which may be attributed to differing inflammatory potentials of these SAA family members. Gutierrez et al. reported, in a metal oxide model of inflammation, that plasma SAA_3_ levels were 1/100th or less the concentration of SAA_1_ [57]. Therefore, lung cells may be more sensitive to SAA_3_ than SAA_1_. Interestingly, loss of ALX/FPR2 caused a decreased neutrophil response to SAA_1_ but an increased neutrophil response to SAA_3_. The SAA family of proteins has also been reported to bind receptors other than ALX/FPR2, including TLR4 [58]. Deguchi et al. reported that SAA_3_ induces peritoneal macrophage migration dependent on TLR4 but independent of ALX/FPR2, while SAA_1_ is known to induce cell migration through ALX/FPR2 [44, 59, 60]. SAA_3_ signaling through TLR4 may explain why neutrophils were increased in FPR2^−/−^ mice dosed with SAA_3_, whereas neutrophils were decreased in FPR2^−/−^ mice dosed with SAA_1_.

In addition to reduced neutrophil recruitment following O_3_ exposure, FPR2^−/−^ mice also had increased neutrophils compared to FPR2^+/+^ mice after FA exposure (Figure 2E). This baseline increase in neutrophil recruitment may be explained by FPR2^−/−^ mice also having increased plasma SAA compared to FPR2^+/+^ mice after FA exposure (Figure 6E). ALX/FPR2 is required for maintaining tissue homeostasis at steady state [61]. Therefore, it is possible that the loss of ALX/FPR2 disrupted homeostasis, resulting in baseline systemic inflammation that was undetected by our current methods but led to increased plasma SAA. Currently, it is unclear whether this affected the pulmonary inflammatory response to O_3_. Future studies will be focused on whether this disruption of homeostasis diminished O_3_‐induced innate immune responses.

This work demonstrates that loss of ALX/FPR2 significantly alters the pulmonary immune response to O_3_ exposure; however, there are several avenues for future investigation. While ALX/FPR2 is most highly expressed in myeloid cells, it is also expressed in lung epithelial cells, the brain, testes, and several other organs [22]. It is possible that the whole‐body knockout disrupts other mechanisms in the body, and future investigation will explore inducible or cell type specific knockouts to more precisely elucidate the role of ALX/FPR2 in the pulmonary immune response to O_3_ exposure. There is also a question of the translatability of this research because of known species differences in SAA_3_. In humans, SAA_3_ is considered a pseudogene because of a single nucleotide insertion leading to a frame shift and early termination of transcription [62]. However, humans express SAA_1_ and SAA_2_ extrahepatically as well, including in monocytes and monocyte‐derived macrophages [63, 64, 65]. Furthermore, this has recently become a subject of discussion because human SAA_3_ was observed to be expressed as a fusion transcript with SAA_2_ and appeared to contribute to inflammation in human cells [66]. Another limitation of this research is that these data focused exclusively on the immune response in males. Sex‐based differences in the pulmonary immune responses to O_3_ have been reported by our group and others [27, 67]. Future studies will evaluate if sex can influence the ALX/FPR2 dependent responses following O_3_.

In conclusion, the present study describes the contribution of ALX/FPR2 and SAA during the pulmonary inflammatory response following O_3_ exposure. The data presented here indicate that ALX/FPR2 mediates SAA‐induced neutrophil recruitment. Ultimately, these findings reveal a novel mechanism of O_3_‐induced inflammation with implications for novel potential therapeutic targets.

## Author Contributions

M.J.Y., R.M.T., R.V., S.R.S., and K.M.G. conceived and designed the research. M.J.Y., N.Z., T.N., K.D.‐R., H.B.L., E.S., G.H., B.S., and R.I.C. performed the research and acquired the data. M.J.Y., S.H., R.M.T., R.V., S.R.S., and K.M.G. analyzed and interpreted the data. All authors were involved in drafting and revising the manuscript.

## Conflicts of Interest

M.J.Y., S.H., R.M.T., S.R.S., and K.M.G. report receiving funding from the National Institutes of Health. S.R.S. reports receiving funding from Metagenics Incorporated and Organic Technologies for work related to PUFAs and SPMs. The authors declare no other conflicts of interest.

## Supporting information


Table S1.

## Data Availability

All data provided within this manuscript will be uploaded to a publicly available database upon acceptance for publication.
