# Supplementary material for: ALX/FPR2 Contributes to Serum Amyloid A‐Induced Lung Neutrophil Recruitment Following Acute Ozone Exposure
Source: FASEB J. 2025 May 27;39(11):e70555. doi: 10.1096/fj.202402865R (PMC12107292; doi:10.1096/fj.202402865R)
Supplement: Supplementary file 1 — Table S1. [file FSB2-39-e70555-s001.docx]

**Supplemental Table**

| **Supplemental Table 1: Detected Lipid Metabolites in Lung Tissue** | | | | | | | | | | | | |
| --- | --- | --- | --- | --- | --- | --- | --- | --- | --- | --- | --- | --- |
| Lipid | Parent Fatty Acid | FPR2^+/+^ + FA | | FPR2^-/-^ + FA | | | FPR2^+/+^ + O_3_ | | | FPR2^-/-^ + O_3_ | | |
|  |  | avg | ± std | avg | ± std |  | avg | ± std |  | avg | ± std |  |
| 11(12)-EpETrE | AA | 1.48 | ± 0.52 | 1.99 | ± 0.48 |  | 2.04 | ± 0.29 |  | 2.07 | ± 0.62 |  |
| 11,12-DiHETrE | AA | 0.35 | ± 0.08 | 0.24 | ± 0.03 | * | 0.35 | ± 0.04 |  | 0.32 | ± 0.07 |  |
| 11-HETE | AA | 275 | ± 50.2 | 215 | ± 24.6 |  | 270 | ± 39.4 |  | 227 | ± 29.1 |  |
| 12(S)-HHTrE | AA | 51.3 | ± 5.36 | 44.8 | ± 3.66 |  | 47.3 | ± 11.8 |  | 52.4 | ± 11.2 |  |
| 12-HETE | AA | 794 | ± 295 | 505 | ± 102 | * | 618 | ± 59.3 |  | 542 | ± 37.8 |  |
| 12-OxoETE | AA | 6.53 | ± 1.19 | 5.59 | ± 2.86 |  | 7.28 | ± 2.12 |  | 5.80 | ± 1.73 |  |
| 12-OxoLTB4 | AA | 0.32 | ± 0.09 | 0.28 | ± 0.06 |  | 0.34 | ± 0.08 |  | 0.37 | ± 0.19 |  |
| 13,14dh-15k-PGD2 | AA | 28.2 | ± 6.46 | 16.6 | ± 4.02 | ** | 24.7 | ± 5.51 |  | 15.5 | ± 3.41 | * |
| 13,14dh-15k-PGE1 | AA | 0.18 | ± 0.02 | 0.16 | ± 0.01 |  | 0.16 | ± 0.04 |  | 0.14 | ± 0.02 |  |
| 13,14dh-15k-PGE2 | AA | 12.7 | ± 0.66 | 11.0 | ± 0.56 |  | 11.7 | ± 0.70 |  | 10.9 | ± 2.07 |  |
| 14(15)-EpETrE | AA | 0.77 | ± 0.24 | 0.92 | ± 0.18 |  | 0.94 | ± 0.19 |  | 0.95 | ± 0.25 |  |
| 14,15-DiHETrE | AA | 0.38 | ± 0.07 | 0.27 | ± 0.06 |  | 0.39 | ± 0.03 |  | 0.37 | ± 0.07 |  |
| 15(R)-PGE1 | AA | 1.59 | ± 0.07 | 1.45 | ± 0.11 |  | 1.61 | ± 0.07 |  | 1.59 | ± 0.49 |  |
| 15-HETE | AA | 68.5 | ± 7.31 | 58.0 | ± 6.20 |  | 64.2 | ± 9.28 |  | 60.7 | ± 10.9 |  |
| 15-keto PGE1 | AA | 0.03 | ± 0.02 | 0.03 | ± 0.03 |  | 0.02 | ± 0.04 |  | 0.05 | ± 0.01 |  |
| 15-keto PGE2 | AA | 2.81 | ± 0.53 | 3.35 | ± 0.34 |  | 2.92 | ± 1.50 |  | 3.08 | ± 1.05 |  |
| 15-keto PGF2a | AA | 11.1 | ± 0.44 | 9.92 | ± 0.74 |  | 10.9 | ± 0.50 |  | 10.3 | ± 2.65 |  |
| 15-oxo LXA4 | AA | 0.02 | ± 0.01 | 0.01 | ± 0.01 |  | 0.02 | ± 0.01 |  | 0.01 | ± 0.02 |  |
| 15-OxoETE | AA | 0.66 | ± 0.11 | 0.56 | ± 0.12 |  | 0.66 | ± 0.10 |  | 0.58 | ± 0.33 |  |
| 20-HETE | AA | 4.61 | ± 1.80 | 5.16 | ± 1.32 |  | 5.46 | ± 0.96 |  | 6.63 | ± 2.16 |  |
| 5(6)-EpETrE | AA | 1.53 | ± 0.44 | 1.53 | ± 0.24 |  | 1.74 | ± 0.27 |  | 1.21 | ± 0.39 |  |
| 5,6-DiHETrE | AA | 0.03 | ± 0.01 | 0.03 | ± 0.01 |  | 0.03 | ± 0.01 |  | 0.02 | ± 0.02 |  |
| 5-HETE | AA | 17.0 | ± 2.94 | 13.1 | ± 3.73 |  | 16.8 | ± 1.71 |  | 15.5 | ± 2.95 |  |
| 5-oxoETE | AA | 2.11 | ± 0.35 | 2.22 | ± 0.37 |  | 2.58 | ± 0.23 |  | 2.46 | ± 0.74 |  |
| 6,15-diketo PGFa | AA | 0.12 | ± 0.06 | 0.09 | ± 0.04 |  | 0.09 | ± 0.02 |  | 0.09 | ± 0.05 |  |
| 6-keto PGE1 | AA | 0.10 | ± 0.03 | 0.07 | ± 0.02 |  | 0.09 | ± 0.03 |  | 0.10 | ± 0.05 |  |
| 6kPGF1a | AA | 37.6 | ± 3.04 | 28.1 | ± 7.02 | * | 32.6 | ± 4.68 |  | 30.3 | ± 6.11 |  |
| 8(9)-EpETrE | AA | 1.03 | ± 0.25 | 1.14 | ± 0.25 |  | 1.06 | ± 0.44 |  | 1.48 | ± 0.79 |  |
| 8,9-DiHETrE | AA | 0.18 | ± 0.10 | 0.19 | ± 0.03 |  | 0.22 | ± 0.10 |  | 0.33 | ± 0.14 |  |
| 8-HETE | AA | 10.4 | ± 1.73 | 9.83 | ± 1.91 |  | 10.9 | ± 1.56 |  | 12.2 | ± 1.66 |  |
| Bicyclo PGE1 | AA | 0.22 | ± 0.04 | 0.17 | ± 0.03 |  | 0.14 | ± 0.06 | † | 0.12 | ± 0.02 |  |
| Bicyclo PGE2 | AA | 0.77 | ± 0.04 | 0.78 | ± 0.13 |  | 0.78 | ± 0.08 |  | 0.77 | ± 0.18 |  |
| iPF-VI | AA | 0.05 | ± 0.03 | 0.02 | ± 0.03 |  | 0.05 | ± 0.03 |  | 0.05 | ± 0.01 |  |
| LXA4 | AA | 0.10 | ± 0.03 | 0.09 | ± 0.03 |  | 0.12 | ± 0.02 |  | 0.11 | ± 0.06 |  |
| PGA2 | AA | 8.39 | ± 0.37 | 8.01 | ± 1.10 |  | 8.49 | ± 0.53 |  | 8.04 | ± 1.93 |  |
| PGD2 | AA | 8.13 | ± 1.39 | 5.65 | ± 2.26 |  | 7.46 | ± 0.72 |  | 4.17 | ± 3.33 |  |
| PGD3 | AA | 0.45 | ± 0.08 | 0.54 | ± 0.03 |  | 0.47 | ± 0.19 |  | 0.49 | ± 0.16 |  |
| PGE1 | AA | 7.00 | ± 0.58 | 5.71 | ± 0.93 | * | 6.62 | ± 0.28 |  | 5.95 | ± 0.68 |  |
| PGE2 | AA | 174 | ± 11.0 | 155 | ± 15.3 |  | 176 | ± 6.10 |  | 164 | ± 25.1 |  |
| PGE3 | AA | 0.88 | ± 0.16 | 1.00 | ± 0.19 |  | 0.97 | ± 0.27 |  | 1.06 | ± 0.36 |  |
| PGF1a | AA | 0.48 | ± 0.07 | 0.41 | ± 0.04 |  | 0.43 | ± 0.10 |  | 0.42 | ± 0.04 |  |
| PGF2a | AA | 11.6 | ± 1.00 | 9.90 | ± 0.81 |  | 10.6 | ± 1.67 |  | 8.68 | ± 0.69 | * |
| PGJ2 | AA | 3.92 | ± 0.22 | 3.56 | ± 0.48 |  | 3.82 | ± 0.14 |  | 3.57 | ± 0.80 |  |
| tetranor 12-HETE | AA | 0.17 | ± 0.06 | 0.15 | ± 0.03 |  | 0.15 | ± 0.04 |  | 0.16 | ± 0.09 |  |
| tetranor PGEM | AA | 0.01 | ± 0.01 | 0.01 | ± 0.00 |  | 0.00 | ± 0.00 |  | 0.01 | ± 0.01 |  |
| TXB2 | AA | 9.03 | ± 0.66 | 6.91 | ± 2.02 |  | 8.13 | ± 0.96 |  | 8.18 | ± 1.71 |  |
| 13(S)-HOTrE | ALA | 2.78 | ± 0.45 | 1.98 | ± 0.64 |  | 2.21 | ± 0.32 |  | 3.03 | ± 1.19 |  |
| 9(S)-HOTrE | ALA | 1.25 | ± 0.28 | 1.31 | ± 0.17 |  | 1.27 | ± 0.32 |  | 1.59 | ± 0.23 |  |
| 9-OxoOTrE | ALA | 0.56 | ± 0.10 | 0.49 | ± 0.07 |  | 0.58 | ± 0.12 |  | 0.63 | ± 0.13 |  |
| 10(11)-EpDPE | DHA | 0.88 | ± 0.32 | 1.13 | ± 0.19 |  | 1.22 | ± 0.10 |  | 1.38 | ± 0.42 |  |
| 10-HDoHE | DHA | 1.71 | ± 0.25 | 1.59 | ± 0.38 |  | 1.90 | ± 0.22 |  | 2.40 | ± 0.53 | † |
| 11-HDoHE | DHA | 1.35 | ± 0.35 | 1.61 | ± 0.40 |  | 1.88 | ± 0.21 |  | 2.29 | ± 0.34 | † |
| 13(14)-EpDPE | DHA | 0.38 | ± 0.16 | 0.57 | ± 0.10 |  | 0.51 | ± 0.13 |  | 0.59 | ± 0.09 |  |
| 13-HDoHE | DHA | 6.16 | ± 1.17 | 6.23 | ± 0.66 |  | 6.98 | ± 1.35 |  | 7.68 | ± 0.59 |  |
| 14-HDoHE | DHA | 24.3 | ± 4.09 | 16.5 | ± 6.85 |  | 22.1 | ± 4.99 |  | 25.5 | ± 8.84 |  |
| 16(17)-EpDPE | DHA | 0.30 | ± 0.10 | 0.41 | ± 0.08 |  | 0.41 | ± 0.05 |  | 0.44 | ± 0.09 |  |
| 16-HDoHE | DHA | 3.61 | ± 0.49 | 4.36 | ± 1.07 |  | 4.88 | ± 0.59 | † | 5.64 | ± 0.79 |  |
| 17-HDoHE | DHA | 2.43 | ± 0.56 | 1.59 | ± 0.46 |  | 2.09 | ± 0.34 |  | 2.37 | ± 0.70 |  |
| 19(20)-EpDPE | DHA | 0.39 | ± 0.11 | 0.49 | ± 0.09 |  | 0.55 | ± 0.05 |  | 0.57 | ± 0.13 |  |
| 19,20-DiHDoPE | DHA | 0.18 | ± 0.01 | 0.16 | ± 0.02 |  | 0.18 | ± 0.01 |  | 0.19 | ± 0.01 | †† |
| 20-HDoHE | DHA | 4.27 | ± 0.82 | 5.21 | ± 0.94 |  | 5.76 | ± 0.72 | † | 6.55 | ± 0.77 | † |
| 4-HDoHE | DHA | 4.58 | ± 1.00 | 5.90 | ± 1.18 |  | 6.56 | ± 0.89 | † | 7.06 | ± 1.11 |  |
| 7(8)-EpDPE | DHA | 0.19 | ± 0.07 | 0.26 | ± 0.04 |  | 0.25 | ± 0.03 |  | 0.28 | ± 0.06 |  |
| 7-HDoHE | DHA | 0.73 | ± 0.14 | 0.83 | ± 0.19 |  | 0.92 | ± 0.10 |  | 1.11 | ± 0.26 |  |
| 8-HDoHE | DHA | 2.00 | ± 0.59 | 2.56 | ± 0.69 |  | 2.62 | ± 0.41 |  | 3.13 | ± 0.43 |  |
| Maresin1 | DHA | 0.05 | ± 0.11 | 0.21 | ± 0.23 |  | 0.39 | ± 0.21 |  | 0.40 | ± 0.45 |  |
| PD1 | DHA | 0.17 | ± 0.10 | 0.14 | ± 0.13 |  | 0.24 | ± 0.16 |  | 0.45 | ± 0.43 |  |
| RvD6 | DHA | 0.05 | ± 0.01 | 0.05 | ± 0.01 |  | 0.07 | ± 0.00 |  | 0.07 | ± 0.02 |  |
| 11(R)-HEDE | EDA | 9.24 | ± 1.65 | 7.97 | ± 1.31 |  | 9.37 | ± 1.97 |  | 9.53 | ± 1.09 |  |
| 15(S)-HEDE | EDA | 0.93 | ± 0.22 | 0.72 | ± 0.17 |  | 0.91 | ± 0.14 |  | 0.92 | ± 0.21 |  |
| 15-OxoEDE | EDA | 0.16 | ± 0.06 | 0.10 | ± 0.06 |  | 0.21 | ± 0.03 |  | 0.16 | ± 0.06 |  |
| 11-HEPE | EPA | 1.98 | ± 0.23 | 1.59 | ± 0.35 |  | 1.70 | ± 0.21 |  | 1.69 | ± 0.16 |  |
| 12-HEPE | EPA | 60.9 | ± 9.32 | 44.0 | ± 14.6 |  | 52.1 | ± 16.9 |  | 57.5 | ± 12.8 |  |
| 14(15)-EpETE | EPA | 0.19 | ± 0.03 | 0.17 | ± 0.07 |  | 0.21 | ± 0.04 |  | 0.16 | ± 0.05 |  |
| 15(S)-HEPE | EPA | 1.52 | ± 0.36 | 0.78 | ± 0.40 | * | 1.07 | ± 0.11 |  | 1.08 | ± 0.54 |  |
| 18-HEPE | EPA | 0.22 | ± 0.05 | 0.17 | ± 0.02 |  | 0.20 | ± 0.04 |  | 0.20 | ± 0.06 |  |
| 5(S),12(S)-DiHETE | EPA | 0.07 | 0.05 | 0.02 | 0.04 |  | 0.02 | 0.03 |  | 0.05 | 0.04 |  |
| 5(S),15(S)-DiHEPE | EPA | 0.12 | ± 0.02 | 0.11 | ± 0.01 |  | 0.12 | ± 0.02 |  | 0.16 | ± 0.07 |  |
| 5-HEPE | EPA | 0.45 | ± 0.13 | 0.26 | ± 0.15 |  | 0.38 | ± 0.05 |  | 0.33 | ± 0.09 |  |
| 8(9)-EpETE | EPA | 0.01 | ± 0.01 | 0.02 | ± 0.01 |  | 0.02 | ± 0.01 |  | 0.01 | ± 0.01 |  |
| 8-HEPE | EPA | 0.23 | ± 0.04 | 0.20 | ± 0.06 |  | 0.19 | ± 0.07 |  | 0.23 | ± 0.03 |  |
| 5(S)-HETrE | GLA | 0.20 | ± 0.04 | 0.17 | ± 0.04 |  | 0.18 | ± 0.02 |  | 0.19 | ± 0.05 |  |
| 8(S)-HETrE | GLA | 3.76 | ± 0.74 | 3.66 | ± 0.90 |  | 4.12 | ± 0.94 |  | 4.95 | ± 1.13 |  |
| 12(13)-EpOME | LA | 2.99 | ± 0.78 | 3.31 | ± 0.41 |  | 3.40 | ± 0.47 |  | 4.01 | ± 1.23 |  |
| 12,13-DiHOME | LA | 0.47 | ± 0.10 | 0.35 | ± 0.11 |  | 0.46 | ± 0.13 |  | 0.41 | ± 0.10 |  |
| 13-HODE | LA | 198 | ± 28.6 | 171 | ± 29.0 |  | 181 | ± 30.0 |  | 207 | ± 41.7 |  |
| 13-OxoODE | LA | 46.4 | ± 10.2 | 44.3 | ± 6.06 |  | 49.1 | ± 8.91 |  | 55.6 | ± 14.9 |  |
| 9(10)-EpOME | LA | 8.91 | ± 3.42 | 10.4 | ± 2.14 |  | 10.3 | ± 1.62 |  | 12.6 | ± 3.76 |  |
| 9,10-DiHOME | LA | 0.52 | ± 0.07 | 0.37 | ± 0.10 | * | 0.46 | ± 0.08 |  | 0.42 | ± 0.09 |  |
| 9-HODE | LA | 27.5 | ± 15.6 | 26.2 | ± 4.77 |  | 22.2 | ± 2.16 |  | 31.3 | ± 8.03 |  |
| 9-OxoODE | LA | 26.0 | ± 8.62 | 24.0 | ± 2.70 |  | 25.3 | ± 8.68 |  | 37.7 | ± 10.4 |  |
| ALX/FPR2 wild type (FPR2^+/+^) or ALX/FPR2 knockout (FPR2^-/-^) male mice 8-12 weeks old were exposed to filtered air (FA) or 1ppm ozone (O_3_) for 3h and necropsied 24h after the start of the exposure. Lung tissue was collected for gas chromatography analysis of lung fatty acids. †p<0.05 between exposures, *p<0.05 between genotypes, n=5-6/group. Avg., Average; Std., Standard Deviation | | | | | | | | | | | | |
